# Evaluation of Hypoglycemic Efficacy of Tangningtongluo Formula, a Traditional Chinese Miao Medicine, in Two Rodent Animal Models

**DOI:** 10.1155/2014/745419

**Published:** 2014-11-05

**Authors:** Long Cheng, Xiang-bao Meng, Shan Lu, Ting-ting Wang, Yue Liu, Gui-bo Sun, Xiao-bo Sun

**Affiliations:** ^1^Key Laboratory of Bioactive Substances and Resources Utilization of Chinese Herbal Medicine, Institute of Medicinal Plant Development, Chinese Academy of Medical Sciences and Peking Union Medical College, Ministry of Education, No. 151, Malianwa North Road, Haidian District, Beijing 100193, China; ^2^Heilongjiang University of Chinese Medicine, Harbin, Heilongjiang 150040, China; ^3^Pharmaceutical College, Harbin University of Commerce, Harbin, Heilongjiang 150076, China; ^4^Xiyuan Hospital, China Academy of Traditional Chinese Medicine, Beijing 100091, China

## Abstract

Traditional Chinese medicines largely lack adequate and scientifically rigorous evidence regarding efficacy and functional mechanisms. The present study was aimed to confirm the hypoglycemic effect of Tangningtongluo (TNTL) formula, a traditional Chinese Miao medicine, in two animal models: high-fat diet and streptozotocin- (STZ-) induced diabetic rats and C57BL/KsJ-db/db diabetic mice. After 4 weeks, TNTL intervention in STZ-induced diabetic rats yielded in significant improvement on the glucose tolerance test. Moreover, the islet histopathology showed that oral TNTL reduced the severity of islet necrosis in pancreases tissue. Compared with diabetic controls, a 12-week TNTL treatment regimen (dosages = 0.9, 1.8, and 3.6 g/kg) in db/db mice significantly decreased fasting glucose and HbA1c. Additionally, oral glucose tolerance in TNTL-treated mice improved significantly, compared with diabetic mice receiving metformin. Finally, tissue histopathology and biochemical index evaluations revealed significant improvement in TNTL-treated mice. Taken together, our results show that TNTL exerted a strong hypoglycemic effect in two diabetic rodent animal models, preserving *β*-cells in the pancreas islet and reducing the risk of diabetic retinopathy and nephropathy.

## 1. Introduction

Type 2 diabetes mellitus (T2DM) is a chronic metabolic disease characterized by dysregulation of glucose and lipid metabolism [1, 2], which mainly linked to abnormal blood insulin levels or insensitivity of target organs to insulin [3]. Insulin resistance or deficiency results in profound dysregulation of glucose metabolism, and elevation in fasting and postprandial glucose levels, which in turn damage many of the body's systems, in particular the blood vessels and nerves [4].

The prevalence of diabetes, especially T2DM, is increasing markedly in China and worldwide [5, 6]. The International Diabetes Federation (IDF) predicts that the global prevalence of diabetes will grow from 382 million in 2013 to 592 million by 2035 [7, 8]. Because T2DM and its complications associate with considerable socioeconomic burden and rising mortality, there is increasing interest in developing strategies to prevent or delay disease progression. Currently, clinical treatment of T2DM relies mainly on western medicines (e.g., sulfonylurea, biguanides, thiazolidinediones, and glycosidase inhibitors) to control hyperglycemia and insulin resistance [9, 10]. Because western drugs cannot fulfill all clinical needs due to accessibility, clinical efficacy, and safety defects, the need for novel and inexpensive drugs and therapies to improve T2DM treatment and reduce the risk of complications has become urgent. Traditional Chinese medicine (TCM) has played an important role in the healthcare system in Asia region, especially in the vast rural areas of China [8, 11]. Thus, searching for new antidiabetic drug from TCM herbal formulae is a low risk and valuable strategy in new drug development [12–14]. Traditional Chinese Miao medicine, a branch of TCM, is practiced widely in southwest China. Tangningtongluo formula (TNTL), an empirical formula of Miao medicine, may possess synergistic antidiabetic effects but currently lacks systematic evidence of its hypoglycemic effect. Therefore, the present study aimed to explore the application value of TNTL from beside to bench.

## 2. Materials and Methods

### 2.1. Chemical Reagents and Plant Material

Streptozotocin (STZ) was purchased from Sigma (St. Louis, MO, USA), and mouse insulin ELISA kits (batch number: 13061201) were purchased from Crystal Chem Company. Rat insulin and C-peptides ELISA kits were obtained from Sweden Mercodia Company. A One-Touch Ultra Blood Glucose Meter and strips (lot number: 3462320) were obtained from Johnson & Johnson Medical Equipment Co., Ltd. (Shanghai, China); total cholesterol (TC), triglyceride (TG), low-density lipoprotein cholesterol (LDL), and high-density lipoprotein cholesterol (HDL) kits were purchased from BHKT Clinical Reagents Co., Ltd. (Beijing, China). We used carboxyl methyl cellulose (0.5%) solution as vehicle. Metformin (Met) and gliclazide (Glic) were suspended in 0.5% carboxyl methyl cellulose solution and used as reference drugs for hypoglycemic activity. Metformin hydrochloride tablets were purchased from Shanghai Squib Pharmaceutical Co., Ltd. (Shanghai, China). Gliclazide tablets were purchased from Tianjin Huajin Pharmaceutical Co., Ltd. (Tianjin, P. R. China). All solvents and other chemicals were of analytical grade.

Plant material was collected from Guizhou and then was botanically authenticated, and a voucher specimen has been deposited in the Miao Medicine Herbarium of Guizhou Bailing pharmaceutical co., Ltd, Guizhou Province, China. This company also provided aqueous extract of the TNTL formula. To obtain the aqueous extract from the herb formula [herba Plantaginis (Cheqiancao) 1.16 kg, kewoluoqu 1.6 kg, Flos Lonicerae (Shanyinghua) 1.11 kg herba Agrimoniae (Xianhecao) 1.11 kg] was cleaned and extracted with boiling water by dynamic maceration twice, every time for 1 h. After filtering, the solution was treated with spray drying to dryness, yielding extract powder (1.0 kg TNTL extract), which was used for evaluation of antidiabetic activity. To reduce the variability of TNTL among different batches, the species, origin, harvest time, medicinal parts, and concocted methods for each component were strictly standardized in GMP condition. Moreover, for the quality control, the fingerprint of TNTL was established by HPLC-UV (supplemental Figure 1 in Supplementary Material available online at http://dx.doi.org/10.1155/2014/745419). In 2014, the preparation of TNTL was authorized by the local drug administration management department (Authorized Number: QYZZ-2014033).

### 2.2. Streptozotocin-Induced Diabetes Mellitus in Rats

Male Sprague Dawley (SD) rats were housed in stainless steel cages and kept under controlled conditions (temperature, 23 ± 2°C; relative humidity, 55 ± 10%; and ventilation, >10 times/hour; 12-hour light/dark cycle). All animals had free access to food and water throughout the acclimation and experimentation periods and were maintained according to Beijing Laboratory Animal Management Regulations. The Animal Management Committee of the Animal Resource Center, Institute of Medicinal Plant Development (IMPLAD), Chinese Academy of Medical Sciences (Beijing, China), reviewed and approved the experiment protocol.

The diabetic rat was developed by high-fat diet (HFD) feeding and intraperitoneal injection of STZ (30 mg/kg BW) [15, 16], whereas the rats in the control group consumed a regular diet without STZ injection. Following 4 weeks of HFD intervention, we injected the rats with STZ (30 mg/kg BW, dissolved in sodium citrate buffer, pH 4.4), followed by a glucose solution (20%, 1.0 g/kg) to prevent initial drug-induced hypoglycemia. STZ-injected animals exhibited hyperglycemia within a few days, as evidenced by the results of a fasting blood glucose test obtained with a blood sugar meter and blood sugar test paper (Johnson & Johnson). We confirmed the diabetic condition by detecting the elevated plasma glucose level 3–5 days after STZ injection. We used nondiabetic rats as controls (Group I) and randomized diabetic rats into five groups (II~VI). Group I and Group II (diabetic controls) received an equal volume of vehicles. Diabetic rats received the antidiabetic agent metformin (Met, 210 mg/kg BW, i.g) (Group III) or gliclazide (Glic, 37 mg/kg BW i.g) (Group IV), or TNTL (0.63 g/kg BW i.g. [Group V] or 1.26 g/kg BW i.g. [Group VI]).

*Experimental Protocol*. We randomly divided DM rats into five groups (eight rats per group). Experimental groups orally ingested TNTL, or metformin, or gliclazide suspended in 0.5% carboxyl methyl cellulose for 28 days, using an intragastric tube daily. We recorded the initial and final body weight of all rats. On day 27, all animals were fasted overnight before receiving a 20% glucose solution (3.0 g/kg) for the oral glucose tolerance test (OGTT). After OGTT, all the animals were anesthetized and sacrificed by cervical decapitation. Blood samples were collected in tubes to detect plasma insulin and C-peptides concentrations. 

*Histopathologic Examination*. To prepare harvested tissue section for examination by light microscopy, we removed the pancreas, preserved it in 10% neutral phosphate-buffered formalin, and processed the tissue by routine paraffin sectioning and staining with hematoxylin and eosin (H&E). Staining was performed according to the manufacturers' instructions. Pathological changes were observed under optical microscope. 

*Biochemical Analysis*. To determine plasma insulin and C-peptide levels, we performed an enzyme-linked immunosorbent assay using commercial kits.

### 2.3. Diabetic Model of C57BL/KsJ-db/db Mice

To confirm hypoglycemic activity, we purchased 6-week-old female C57BL/KsJ-db/db mice as DM model [17–19] from SLAC Laboratories Animal Co., Ltd. (Shanghai, China). Animals were housed under controlled conditions (temperature, 23° ± 2°C; relative humidity, 50% ± 10%; 12 h light/dark cycle) and allowed free access to standard diet. All mice were acclimatized for 2 weeks prior to the initiation of the test and maintained according to Beijing Laboratory Animal Management Regulations. The experiment protocol was reviewed and approved by the Animal Management Committee of Animal Resource Center, IMPLAD, Chinese Academy of Medical Sciences. 

*Experimental Protocol*. We randomly divided db/db mice into five groups, each containing twelve mice. C57BL mice group (Group 1, normal control group) and db/db diabetic controls (Group 2) received an equal volume of vehicle. Positive controls (Group 3) received the antidiabetic agent, metformin (Met, 120 mg/kg BW i.g.). TNTL-treated mice received 3.6 g, 1.8 g, or 0.9 g/kg BW i.g. (Groups 4, 5, and 6, resp.). All mice received the appropriate intervention daily for 12 weeks, and we measured weight, food, and water intake every week. To determine fasting blood glucose levels, we drew blood samples from the tail vein of all mice every week. After placing fresh blood (approximately 50 *μ*L) on duplicate test strips, we used a validated One-Touch Basic Glucose Monitoring System to determine the glucose content. In week 12, all animals were fasted for 5 hours and anesthetized with diethyl ether. After injecting fluorescein (20 *μ*L) into the tail vein of mice, we used optical coherence tomography (OCT) and fluorescein angiography (FA) to detect early diabetes-induced changes in retinal thickness and microvasculature with a Phoenix Micron IV In Vivo Retinal Imaging Microscope (Phoenix Research Labs, Pleasanton, CA, USA). On day 86, all animals were fasted overnight and then given a 20% glucose solution (3.0 g/kg) prior to OGTT. After OGTT, all animals were anesthetized and sacrificed by cervical decapitation. Blood samples were collected for the biochemical analysis. 

*Biochemical Analysis*. We centrifuged blood samples at 3000 ×g for 15 min and then removed and stored the plasma (−60°C) for further analysis. We used commercial kits to detect plasma insulin levels by enzyme-linked immunosorbent assay. We used commercial kits and a standard assay method to estimate the plasma lipid profile (TG, TC, HDL, and LDL) liver and kidney function indexes. 

*Histopathologic Examination*. To prepare harvested tissue for examination by light microscopy, we removed and preserved pancreas, kidney, and liver sections in 10% neutral phosphate-buffered formalin and processed them by routine paraffin sectioning and staining with hematoxylin and eosin (H&E). Staining was performed according to the manufacturers' instructions. Pancreas, fundus oculi, kidney, and liver tissue sections were observed under a light microscope.

### 2.4. Statistical Analysis

Statistical analysis was performed with SPSS software, version 11.5 (Armonk, NY, USA). All data were expressed as mean ± SD. Comparisons between groups were analyzed by one-way ANOVA, and group comparisons were analyzed using the Student-Newman-Keuls test. A value of *P* < 0.05 or less was considered statistically significant.

## 3. Results

### 3.1. TNTL Improved Glucose Tolerance in STZ-Induced Diabetic Rats

**Figure 1:**
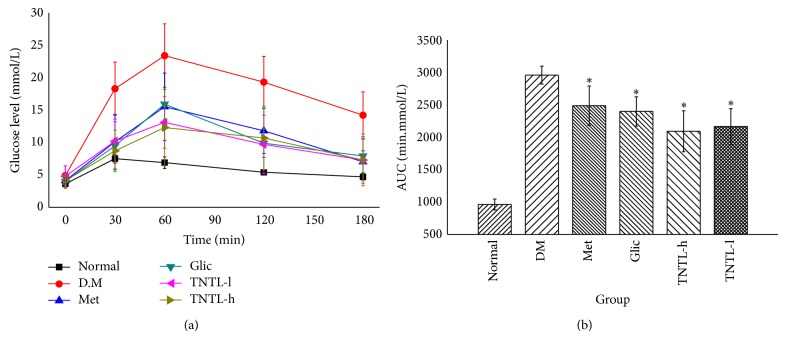
The results of OGTT in STZ-induced diabetic rats. TNTL reduced the blood glucose level (a) after oral high dose of glucose and decreased the area under the glucose response curve (b). Normal: SD without any treatment, DM: STZ-induced diabetic rats without intervention, Met: STZ-induced diabetic rats treated with metformin, and TNTL-h, l: STZ-induced diabetic rats treated with 1.26 g and 0.63 g/kg b.w., respectively. ^*^ *P* < 0.05 compared with DM group.

STZ-induced DM rats (Groups VI and V) receiving oral TNTL (1.26 and 0.63 g/kg, resp.) for 4 weeks revealed significantly suppressed the elevated plasma glucose at 30, 60, 120, and 180 min after ingestion of a single high dose of glucose (Figure 1(a)), as well as decreased area under the glucose response curve (AUC 2, 094.3 ± 316.2, 2, 166.5 ± 716.3 min·mmol/L, Figure 1(b)), compared to diabetic controls Group II (2, 958.9 ± 138.7 min·mmol/L). As expected, incremental plasma glucose levels and AUC in Groups III (210 mg/kg) and IV (37 mg/kg) also decreased significantly compared to Group II. Plasma insulin and C-peptide decreased significantly in Group II compared to Group I. We observed no significant differences in serum insulin and C-peptide concentrations between TNTL- (1.26 and 0.63 g/kg) and metformin- (210 mg/kg), gliclazide- (37 mg/kg) treated rats, and untreated STZ-induced DM groups.

### 3.2. TNTL Alleviated Pancreas Lesion Severity in STZ-Induced Diabetic Rats

**Figure 2:**
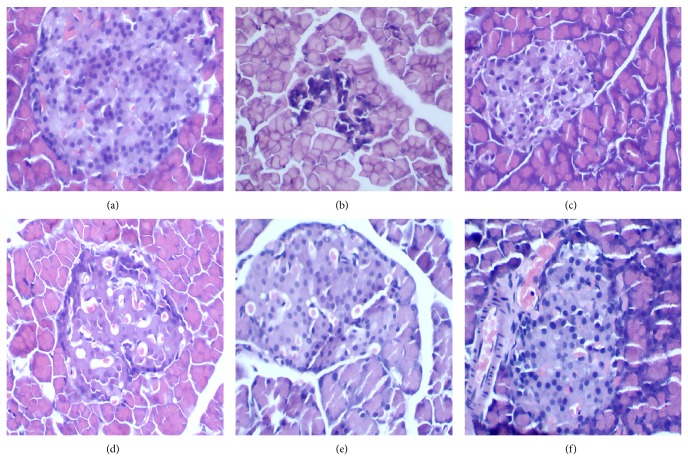
The pathological examination result of pancreatic islets in STZ-induced diabetic rats. (a) Pancreatic islet from a normal mouse. (b) Pancreatic islet from STZ-induced diabetic rats with no treatment, severe degranulation of most *β*-cells in this islet. (c) and (d) Pancreatic islet from STZ-induced rats treated with Met and Glic, respectively. (e) and (f) Pancreatic islet from STZ-induced rats treated with TNTL (1.26 and 0.63 g/kg b.w., resp.).

Figure 2 shows the pathology of STZ-induced DM rat pancreas after 4 weeks of intervention. Pathological features of the pancreas in Group I showed no histopathological changes in the architecture of normal islet cells (Figure 2(a)). The STZ-induced DM group rats exhibited islet degeneration and a definitive loss of *β*-cells (Figure 2(b)), whereas islet architecture was preserved in TNTL-treated rats (Figures 2(e) and 2(f), resp.), with less necrosis of *β*-cells. Thus, TNTL in the pancreas ameliorated STZ-induced destruction of *β*-cells and the degree of inflammation. As expected, gliclazide (37 mg/kg) treatment yielded a moderate protective effect.

### 3.3. Lowering Fasting Blood Glucose Level and Improvement Glucose Tolerance in db/db Mice

**Figure 3:**
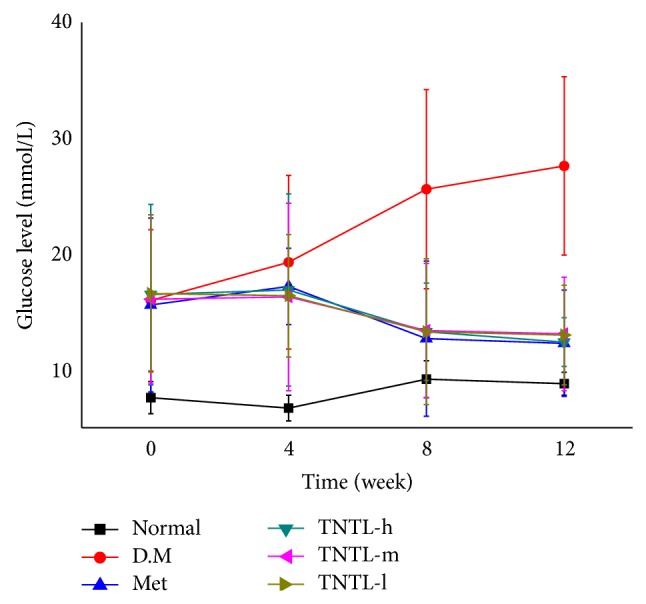
Fasting blood glucose levels of db/db mice. Fasting blood glucose levels of db/db mice with no treatment or with metformin and TNTL (TNTL-h, m, l = 3.6 g, 1.8 and 0.9 g/kg BW, resp.) oral administration. TNTL exhibits hypoglycemic effect with sustained medication. Normal: C57BL, DM: db/db mice with untreated, Met: db/db mice treated with metformin, and TNTL-h, m, l: db/db mice treated with 3.6 g, 1.8 and 0.9 g/kg b.w., respectively.

**Figure 4:**
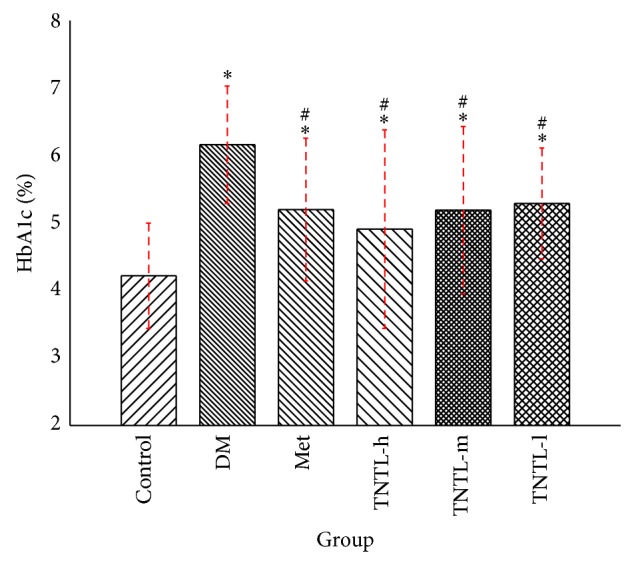
HbA1c levels in db/db mice. HbA1c levels of control (C57BL), DM (db/db mice with untreated), Met (treated with metformin), or TNTL-treated (TNTL-h, m, l = 3.6 g, 1.8 and 0.9 g/kg b.w., resp.) oral administration. ^#^ *P* < 0.05 compared with DM group. ^*^ *P* < 0.05 compared with control group.

The intake of TNTL (3.6, 1.8 and 0.9 g/kg b.w) or metformin (120 mg/kg) did not significantly influence body weight, food and water intake, or feed efficiency ratio in db/db mice. At the age of 6 weeks, the serum glucose level of db/db mice is significant hyperglycaemic. As Figures 3 and 4 show, the intake of TNTL resulted in a significant dose-related decrease in fasting blood glucose and HbA1c. As expected, metformin significantly lowered the levels of blood glucose and HbA1c (Figures 3 and 4) in db/db mice.

**Figure 5:**
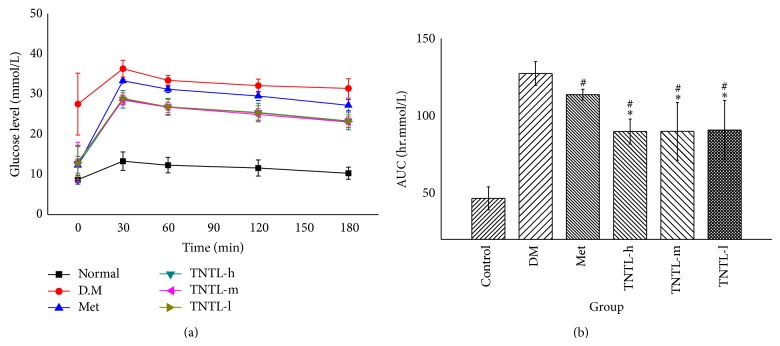
OGTT in db/db mice. TNTL reduced the blood glucose (a) after oral high dose of glucose and decreased the area under the glucose response curve (b). As expected, metformin significantly lowered the AUC in db/db mice compared with diabetic model. Furthermore, TNTL (TNTL-h, m, l = 3.6 g, 1.8 and 0.9 g/kg b.w., resp.) administration significantly lowered the AUC in db/db mice, compared with metformin. Control: C57BL, DM: db/db mice with untreated, Met: db/db mice treated with metformin, and TNTL-h, m, l: db/db mice treated with 3.6 g, 1.8 and 0.9 g/kg b.w., respectively. ^#^ *P* < 0.05 compared with DM group. ^*^ *P* < 0.05 compared with metformin group.

In the oral glucose tolerate test, the consumption of the TNTL and metformin decreased incremental plasma glucose levels (shown in Figure 5(a)). The AUCs in the TNTL (89.8 ± 8.1, 89.9 ± 18.7, 90.8 ± 19.2 hr·mmol/L) and metformin groups (113.7 ± 3.5 hr·mmol/L) were significantly decreased, compared to the diabetic model group (127.3 ± 7.7 hr·mmol/L, shown in Figure 5(b)).

### 3.4. Effect of TNTL on Biochemical Analyses in db/db Diabetic Mice

**Figure 6:**
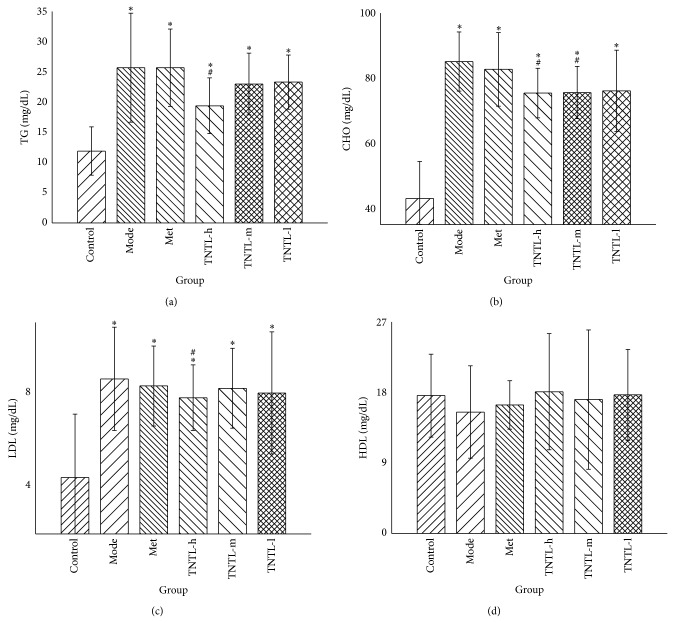
Lipid profile in db/db mice. The TNTL (TNTL-h, m, l means the dose of 3.6 g, 1.8 and 0.9 g/kg b.w., resp.) administration leads to reductions in plasma levels of triglycerides (a), total cholesterol (b), and low-density lipoprotein (c), respectively, compared to the model group. Mice treated with TNTL (3.6 g/kg) showed significant reductions in total cholesterol (TC), CHO, and low-density lipoproteins (LDL) (25%, 11%, and 10%, resp.), compared to controls. Control: C57BL, DM: db/db mice with untreated, Met: db/db mice treated with metformin, and TNTL-h, m, l: db/db mice treated with 3.6 g, 1.8 and 0.9 g/kg b.w., respectively. ^#^ *P* < 0.05 compared with DM group. ^*^ *P* < 0.05 compared with control group.

**Figure 7:**
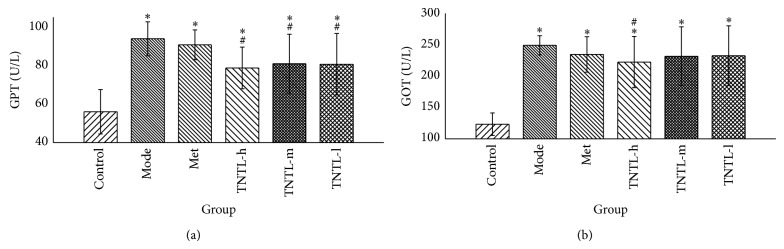
Liver function in db/db mice. TNTL (TNTL-h, m, l = 3.6 g, 1.8 and 0.9 g/kg b.w., resp.) administration resulted in reduced plasma levels of GPT (a) and GOT (b), compared to DM controls. The TNTL groups (3.6 g/kg) show significantly reduced GTP and GOP (16% and 11%, resp.), compared to DM controls. Control: C57BL, DM: db/db mice with untreated, Met: db/db mice treated with metformin, and TNTL-h, m, l: db/db mice treated with 3.6 g, 1.8 and 0.9 g/kg b.w., respectively. ^#^ *P* < 0.05 compared with DM group. ^*^ *P* < 0.05 compared with control group.

The lipid profiles (TC, LDL, and TG) increased significantly, indicating dyslipidemia in db/db diabetic mice. Compared to the diabetic controls (Group 2), plasma concentrations of TC, TG, and LDL decreased significantly in Group 4 (3.6 g/kg) (11%, 25%, and 10%, resp.) (Figure 6). Glutamic oxaloacetic transaminase (GOT) and glutamic pyruvic transaminase (GPT) increased remarkably, suggesting abnormal liver function. GOT and GPT decreased significantly in Group 4 (3.6 g/kg) (16% and 11%, resp.), compared to the model group (shown in Figure 7).

**Figure 8:**
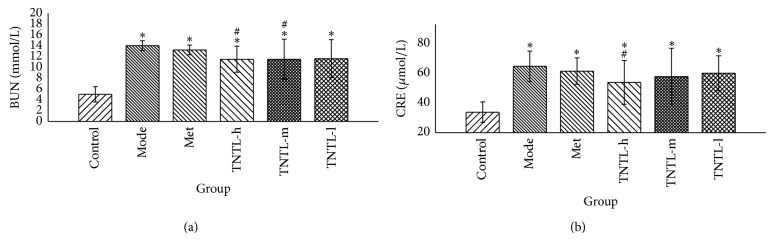
Renal function in db/db mice. The TNTL (TNTL-h, m, l = 3.6 g, 1.8 and 0.9 g/kg b.w., resp.) administration resulted in reduced plasma levels of BUN (a) and CRE (b), compared to the model group. The TNTL groups (3.6 g/kg) have significant reductions in levels of BUN (a) and CRE (b), compared to Met group. The TNTL groups (3.6 g/kg) have significant reductions in levels of BUN and CRE by 18% and 10%, respectively, compared to the model group. Control: C57BL, DM: db/db mice with untreated, Met: db/db mice treated with metformin, and TNTL-h, m, l: db/db mice treated with 3.6 g, 1.8 and 0.9 g/kg b.w., respectively. ^#^ *P* < 0.05 compared with DM group. ^*^ *P* < 0.05 compared with control group.

Compared to Group 1, blood urine nitrogen (BUN) and creatinine (CRE) increased significantly in Group 2. BUN and CRE decreased 18% and 11%, respectively, in Group 4 (3.6 g/kg), compared to Group 2 (Figure 8). Serum insulin did not differ significantly between Groups 3–6 and controls (Group 2).

### 3.5. Effect of TNTL on Retinal Imaging in db/db Diabetic Mice

**Figure 9:**
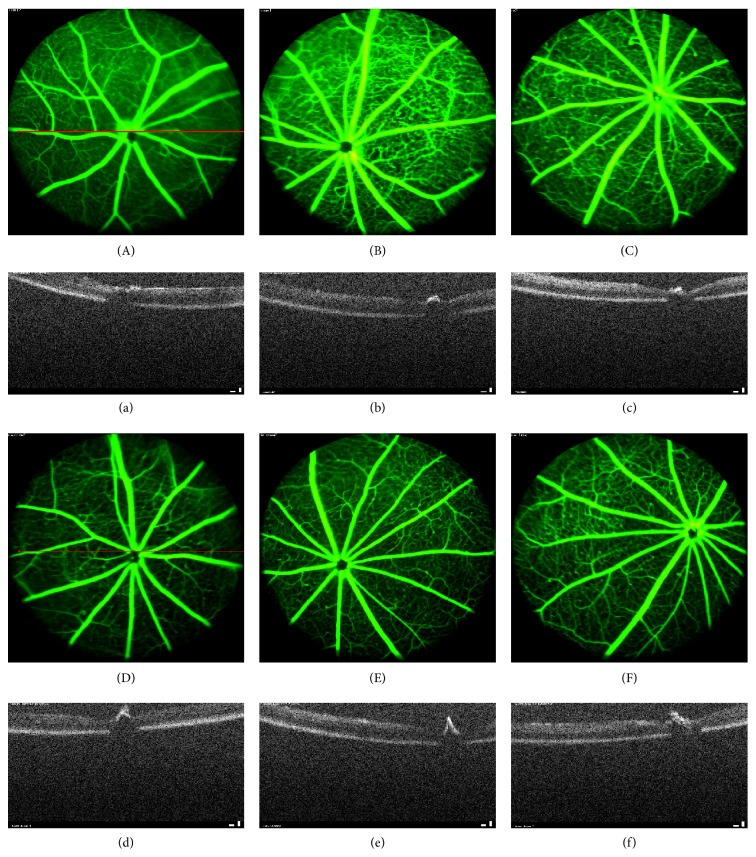
Retinal imaging by OCT and FA in db/db diabetic mice. (A)–(F) Results of examination of the fundus by fluorescein angiography (FA), (a)–(f) Results of retinal imaging by optical coherence tomography (OCT). The OCT result shows the optic nerve fiber layer of db/db mice thinned (b), compared with the nondiabetic group (a). The thinning of optic nerve fiber layer was mildly reversed by the TNTL treatment ((d), (e), and (f) at the dose of 3.6 g, 1.8 and 0.9 g/kg b.w., resp.). There is no alleviation in the Met treatment mice (c) for the optic nerve fiber layer thickness. FA shows that there are numerous vascular calibers (pathological neovascularization) in the db/db mice fundus (B). The TNTL treatment ((D), (E), and (F) at the dose of 3.6 g, 1.8 and 0.9 g/kg b.w., resp.) can significantly reduce the density of vascular calibers in fundus oculi; nevertheless, the positive group (C) shows no significant alleviation.

We used optical coherence tomography (OCT) and fluorescein angiography (FA) to detect progressive neural retinal pathology in animal models of retinal degeneration. OCT revealed thinning of the optic nerve fiber layer in db/db mice, compared with the nondiabetic group. TNTL mildly reversed such thinning (Figures 9(d), 9(e), and 9(f)), compared with untreated db/db mice group (Figure 9(b)). Met did not alleviate layer thickness of the optic nerve fiber (Figure 9(c)). FA revealed numerous vascular calibers (i.e., pathological neovascularization) in the fundus of db/db mice (Figure 9(b)). As Figures 9(d), 9(e), and 9(f) show, the TNTL treatment can significantly reduce the density of vascular calibers in fundus oculi; nevertheless, the positive group (Met treatment) shows no alleviation in pathological neovascularization (shown in Figure 9(c)).

### 3.6. Histological Profiles of the Effect of TNTL on Lesion Severity in db/db Diabetic Rats

**Figure 10:**
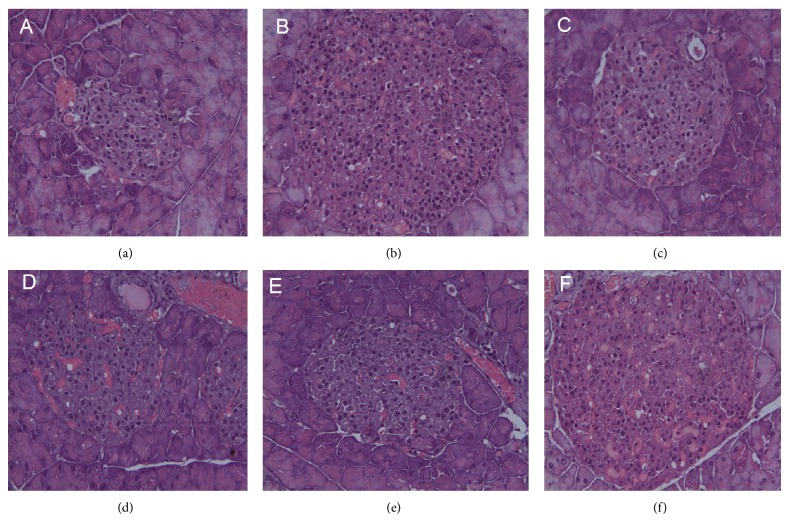
Pathological examination of pancreatic islets in db/db mice. (a) Pancreatic islet from a normal mouse. (b) Pancreatic islet from db/db mouse with no treatment shows severe degranulation of most *β*-cells in this islet. (c) Pancreatic islet from db/db mouse treated with metformin. (d), (e), and (f) Pancreatic islet from db/db mouse treated with TNTL at the dose of 3.6 g, 1.8 and 0.9 g/kg b.w., respectively.

Figure 10 shows the pancreatic pathology of db/db mice after 12 weeks' treatment. Pancreas pathological features of normal control group showed no histopathological changes with normal islet cells architecture (Figure 10(a)). Pancreas of diabetic mice showed hyperplasia of islets but decreased *β*-cell mass due to the apoptosis of *β*-cells (Figure 10(b)). Pancreas pathology of diabetic mice treated with TNTL and metformin is shown in Figures 10(d), 10(e), 10(f), and 10(c), respectively. These results indicated that destruction of islet and the degree of inflammation were ameliorated by TNTL.

**Figure 11:**
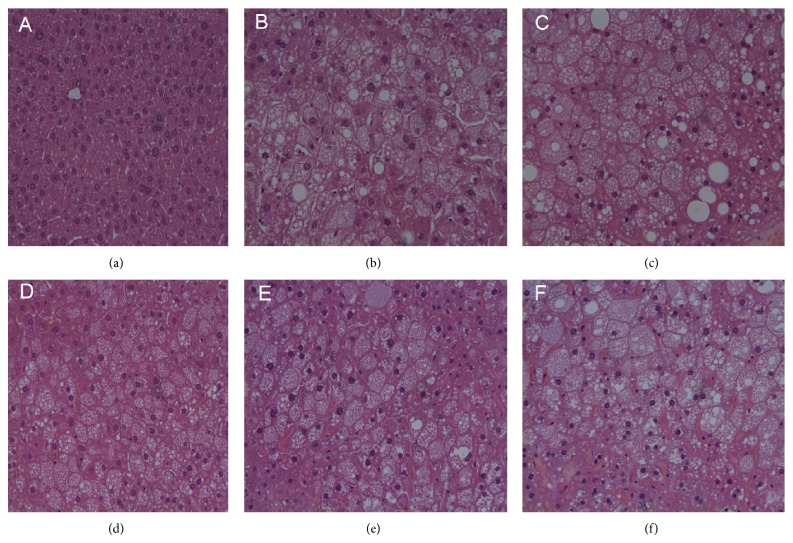
The pathological examination of liver tissue in db/db mice. (a) Hepatic cellular architecture from a normal mouse. (b) Hepatic cellular architecture from db/db mouse with no treatment shows ballooning degeneration and fatty degeneration of hepatocytes. (c) Microscopic view of degenerated hepatocytes from metformin-treated db/db mouse. (d), (e), and (f) Alleviation of hepatocytes degeneration from pancreatic islet from db/db mouse treated with TNTL at the dose of 3.6 g, 1.8 and 0.9 g/kg b.w., respectively.

Pathological examination of normal mouse livers revealed normal cellular architecture, with distinct hepatic cells and sinusoidal spaces (Figure 11(a)). H&E-stained slides from nontreated DM mice (Group 2) exhibited ballooning and fatty degeneration of hepatocytes. The injuries are clearly alleviated by TNTL (Groups 4–6, Figures 11(d), 11(e), and 11(f)), compared to Group 2 (Figure 11(b)). Histological profiles of liver tissue were consistent with the changes of GOP and GTP. Thus, TNTL treatment reduced GOP and GTP and relieved the liver lesion, indicating a significant liver protective effect.

**Figure 12:**
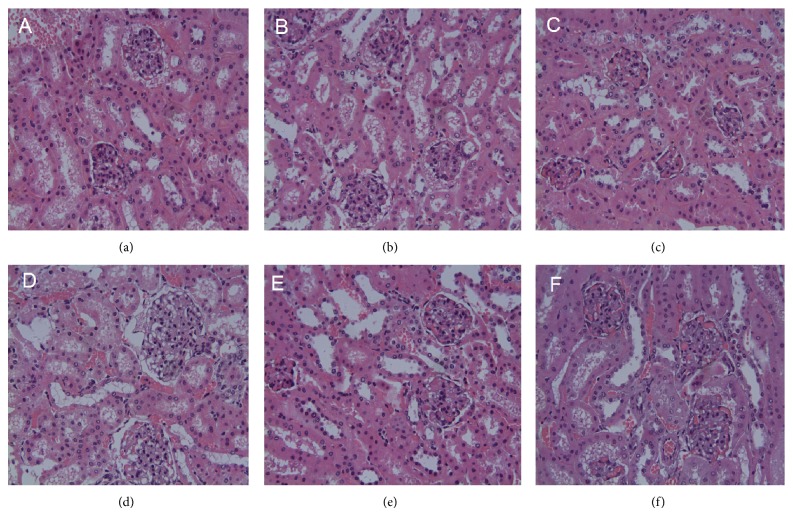
Pathological examination of renal tubule in db/db mice. (a) Renal tubule from a normal mouse. (b) Edema and degeneration of renal tubular epithelial cell from db/db mouse with no treatment. (c) Edema and degeneration of renal tubular epithelial cell from metformin-treated db/db mouse. (d), (e), and (f) Alleviation of degeneration of renal tubular epithelial cell from TNTL-treated db/db mouse at the dose of 3.6 g, 1.8 and 0.9 g/kg b.w., respectively.

H&E-stained slides of diabetic mouse tissue exhibited edema and degenerating renal tubular epithelial cells (Figure 12). Kidney injury decreased somewhat in Groups 4–6, compared with the Group 2. Histological profiles of kidney tissue concurred with the changes in renal function, indicating a renal protective effect.

**Figure 13:**
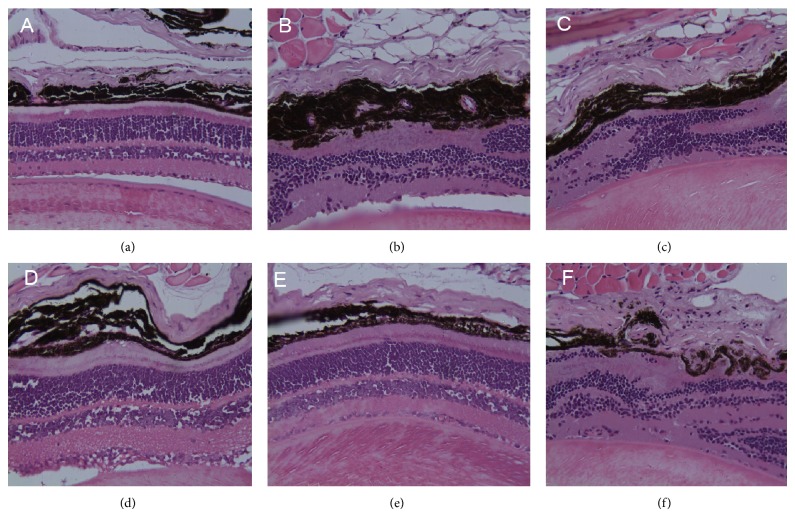
Pathological examination of fundus in db/db mice. (a) Fundus oculi from a normal mouse. (b) Retinopathy from db/db mice, featuring neuron loss and glioses in the ganglion cell layer. (c) Retinal lesions from metformin-treated db/db mouse. (d), (e), and (f) Mildly alleviated retinal lesions in the TNTL-treated db/db mouse at the dose of 3.6 g, 1.8 and 0.9 g/kg b.w., respectively.

H&E-stained slides from db/db mice exhibited retinopathy, characterized by neuron loss and glioses in the ganglion cell layer (Figure 13) whereas TNTL intervention ameliorated such lesions, compared with Group 2. The metformin-treatment did not significantly alleviate retinopathy (Group 3). Histological profiles of retinal lesions concurred with the changes in retinal thickness and microvasculature, as revealed by OCT and FA, indicating a significant protective effect.

## 4. Discussion

In 2005, the traditional medicine of the Miao community (Guizhou, China) was selected by the Harmony List Jury of UNESCO as the 2005 best cultural practice contributing to sustainable development [20]. Traditional Chinese medicine has been applied by Chinese people from ancient times, which is still in common use in China, especially with the highest prevalence of use in rural areas, for its characteristics of low cost, simplicity, accessibility, and efficacy [15–17, 20]. But the efficacy has been challenged in recent years. For most conditions, as empirical medicine, there is not enough rigorous scientific evidence to know whether and how TCM methods work for the disease condition [15–18, 20]. It is urgent and necessary to systematically evaluate the efficacy and safety of traditional Miao medicine.

We demonstrated here for the first time that TNTL shows an antidiabetic effect and reduction in the risk of microvascular complications of DM in diabetic animal models, as evidenced by glucose-lowering activity and the histopathological observation. In the STZ-induced hyperglycemic rats, diabetes arises from the destruction of pancreatic *β*-cells due to STZ injection [21]. In the present study, TNTL treatment improved glucose tolerance and insulin resistance. Subsequent histological analysis of pancreatic tissue from STZ-injected rats revealed islet shrinkage as well as degenerative and necrotic changes. Moreover, TNTL treatment alleviates islet destruction caused by STZ. Our results show that TNTL ameliorates STZ-induced pancreatic *β*-cell damage and lowers plasma glucose levels. C57BL/KsJ-db/db mice are spontaneously hyperinsulinemia, hyperglycaemia, and insulin resistant after 6–8 weeks of age [22–25], which are commonly and extensively used for the investigation of T2DM [26]. Our results show that the fasting glucose and HbA1c levels decreased significant by the treatment of TNTL at the doses of 0.9, 1.8, and 3.6 g/kg, compared with diabetic model group. Impaired hepatic function may result in the dyslipidosis and hyperglycaemia. The biochemical analyses and histological profile indicated that TNTL can alleviate the liver degeneration and regulate the glucose and liposome metabolism.

Deduced *β*-cell mass, fat deposition into the islets, and deposition of intraislet amyloid occur commonly in human end-stage diabetes [27]. In contrast, the pancreas sections of the diabetic rats examined here showed alterations such as islets shrinkage, cellular swelling, *β*-cell vacuolation, and apoptosis, similar to previous findings [28]. The degeneration of the islets with *β*-cell loss is a significant lesion after insulin resistance [27]. The islet atrophy through *β*-cell loss that remains a thickened layer of peripheral cells (non-*β*) led to the progression of T2DM [17]. TNTL and metformin noticeably lessened the extensiveness of such injuries in T2DM rats. This effect of TNTL is important because cell necrosis is an irreversible process, whereas a good glycemic-control agent can help reverse cell degeneration and enable normal function. We show here that the TNTL mechanism in diabetic mice may relate to improved insulin resistance and *β*-cell preservation in the pancreas islet of rodents.

## 5. Conclusion

In summary, our results suggest that TNTL treatment exerts hypoglycemic activity, improves lipid metabolism, preserves *β*-cells in the pancreas islet, and reduces the risk of microvascular complications of DM. Thus, TNTL may prove beneficial in preventing the progression of T2DM. The present findings may also provide important scientific evidence supporting TNTL formula as an alternative approach for the management of T2DM.

## Supplementary Material

The fingerprint of TNTL with HPLC-UV.
The fingerprint was applied for the TNTL quality control. In the fingerprint, seven main peaks were identified and quantified by the spectral feature of UV and peak areas. The seven main peaks were 1.Chlorogenic acid, 2.Luteoloside, 3.Rutin, 4.Quercetin, 5.Kaempferol, 6.Plantamajoside.

## List of Abbreviations

TNTL: Tangningtongluo formula
TCM: Traditional Chinese medicine
db/db: C57BL/KsJ-db/db
T2DM: Type 2 diabetes mellitus
IDF: International Diabetes Federation
STZ: Streptozotocin
HFD: High-fat diet
HbA1c: Glycated haemoglobin A1c
OCT: Optical coherence tomography
FA: Fluorescein angiography
TC: Total cholesterol
TG: Triglyceride
LDL: Low-density lipoprotein
HDL: High-density lipoprotein
Met: Metformin
Glic: Gliclazide
HFD: High-fat diet
OGTT: Oral glucose tolerance test
BUN: Blood urine nitrogen
CRE: Blood creatinine.
