# Supplementary material for: Evaluation of Hypoglycemic Efficacy of Tangningtongluo Formula, a Traditional Chinese Miao Medicine, in Two Rodent Animal Models
Source: J Diabetes Res. 2014 Nov 5;2014:745419. doi: 10.1155/2014/745419 (PMC4238278; doi:10.1155/2014/745419)
Supplement: Supplementary file 1 — The fingerprint of TNTL with HPLC-UV. The fingerprint was applied for the TNTL quality control. In the fingerprint, seven main peaks were identified and quantified by the spectral feature of UV and peak areas. The seven main peaks were 1.Chlorogenic acid, 2.Luteoloside, 3.Rutin, 4.Quercetin, 5.Kaempferol, 6.Plantamajoside. [file 745419.f1.pdf]

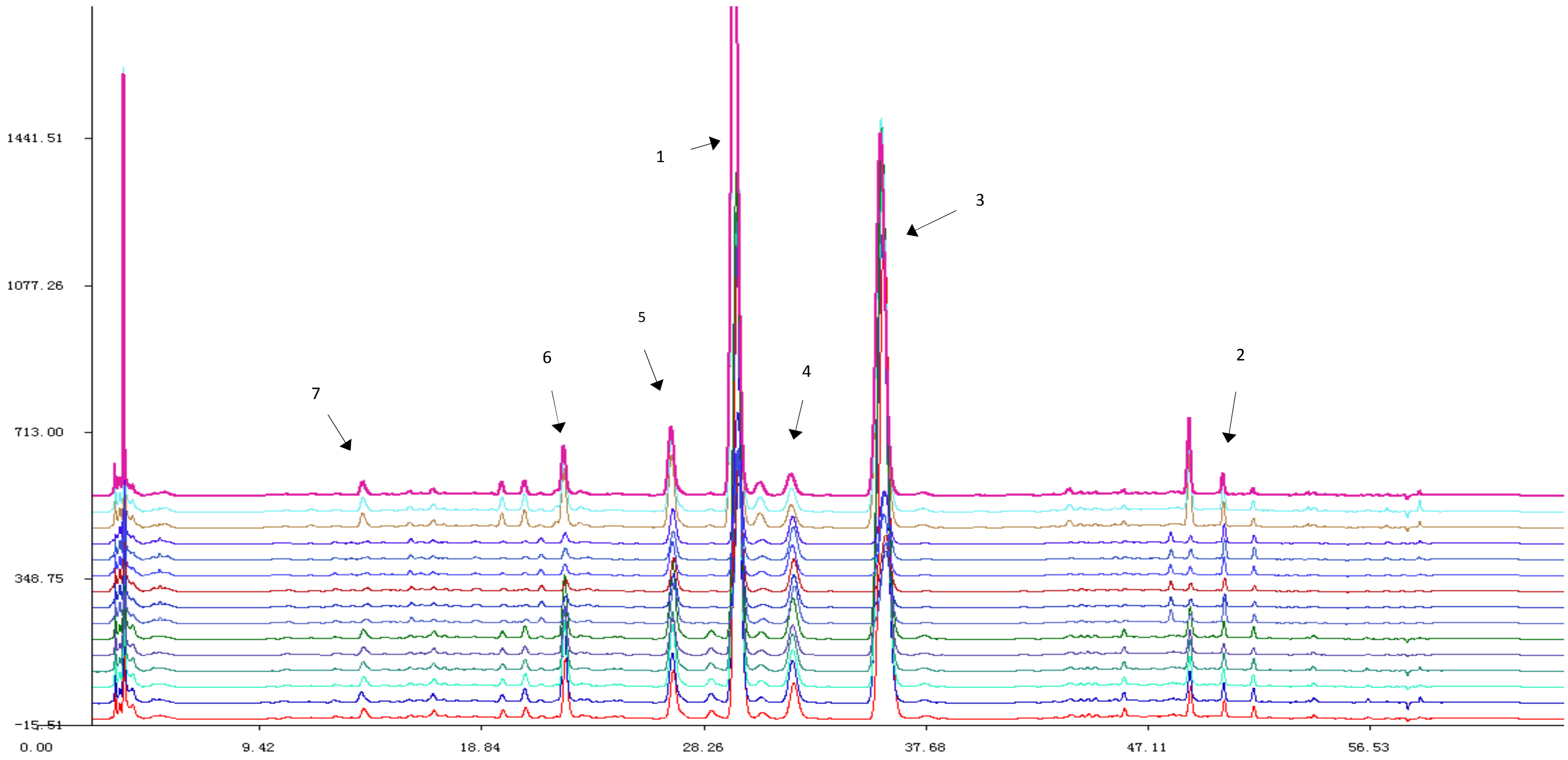

Supplement Figure 1. The HPLC-UV fingerprint of TNTL.

The UV detector wavelength was set at 360 nm. The main peaks were identified and quantified by the spectral feature of UV and peak areas.

1.Chlorogenic acid, 2.Luteoloside, 3.Rutin, 4.Quercetin, 5.Kaempferol, 6.Plantamajoside.
